# Investigation of association between clinically significant prostate cancer, obesity and platelet to-lymphocyte ratio and neutrophil -to-lymphocyte ratio

**DOI:** 10.1186/s12894-024-01617-2

**Published:** 2024-10-16

**Authors:** Johanna Dahan, Jehonathan Pinthus, Guila Delouya, Daniel Taussky, Emmanuelle Duceppe, Amanda de Jesus, Darryl Leong

**Affiliations:** 1 Department of Radiation Oncology, Centre hospitalier de l’Université de Montréal, 1000 rue St Denis, Montréal, QC H2X 0C1 Canada; 2 Department of Surgery, Juravinski Cancer Center/Hamilton Health Sciences, McMaster University, Hamilton, Canada; 3 Department of Surgery, Division of Urology, McMaster University, St. Joseph’s Healthcare, Hamilton, Canada; 4 Department of Medicine, Centre hospitalier de l’Université de Montréal, Montréal, Canada; 5 Population Health Research Institute, McMaster University, Hamilton, Canada; 6 Departments of Medicine and Health Research Methods, Evidence, and Impact, McMaster University, Hamilton, Canada

**Keywords:** Prostate cancer, Obesity, Inflammation, NLR

## Abstract

**Introduction:**

Several blood markers of inflammation are elevated in prostate cancer (PCa) and have prognostic value. Little is known about the relationship between these markers, PCa, and other factors associated with chronic inflammation, such as smoking and obesity. We analyzed the interaction between neutrophil and platelet counts indexed to lymphocyte count (NLR and PLR, resp.) and clinically significant PCa (csPCa), accounting for the potential confounding factors of systemic inflammation.

**Methods:**

NLR and PLR were evaluated in a multicenter prospective study in 443 patients. CsPCa was defined as a Gleason ≥ 4 + 3. Differences between patients with csPCa and non-csPCA were evaluated using the chi-square test, analysis of variance or the Kruskal-Wallis test. Multivariable logistic regression analysis adjusted for smoking, hypertension, diabetes, and cardiovascular disease, and in separate models, either body mass index or waist-to-hip ratio was used to characterize the relationship between inflammation and csPCa.

**Results:**

None of the factors such as plateletcrit, NLR, and PLR were significantly different between patients with csPCa or non-significant PCa. After adjustment, there was no association between PLR, NLR, plateletcrit or platelet count and csPCa. In an exploratory analysis, there was no association between markers of inflammation and PSA levels > 10 ng/mL. When testing different NLR cutoffs to predict csPCa in ROC analysis, none reached a clinically meaningful value.

**Conclusion:**

In contrast to previous studies, we found no significant association between easily available blood markers of inflammation and indices of PCa aggressiveness. Further research is required to determine whether inflammation promotes PCa. (ClinicalTrials.gov: NCT03127631. Date of registration: April 25, 2017.

**Supplementary Information:**

The online version contains supplementary material available at 10.1186/s12894-024-01617-2.

## Introduction

The association between obesity and prostate cancer (PCa) is complex, because obesity is associated at the same time with more aggressive cancer, while at the same time obesity is associated with a decrease in less aggressive cancer [1, 2]. Obesity causes chronic low-grade inflammation which in turn is associated with PCa [3, 4]. Inflammation can change the tumor-microenvironment in PCa and attract different immune-cells and therefore modify the immune-response [5]. Cytokines, such as interleukin (IL)-6, associated with inflammation, demonstrate an important role in the regulation of PCa [6]. Platelets are important in that they secrete inflammatory mediators such as vascular endothelial growth factor (VEGF) and platelet-derived growth factor (PDGF) that themselves influence cancers [7] .

There are several widely available blood markers of inflammation that are known to be elevated in PCa and have a prognostic value [8, 9]. One is the neutrophil-to-lymphocyte ratio which is also a prognostic factor in PCa. A recently published a narrative review showed that NLR as well as the platelet-to-lymphocyte ratio (PLR) can serve as independent prognostic factors in curatively treated PCa [10]. Furthermore, a meta-analysis of patients with metastatic castration-resistant PCa showed that both NLR and PLR were effective prognostic biomarkers [11].

Very little is known about the direct interaction between blood markers of inflammation, such as PLR and NLR, PCa and factors associated with chronic inflammation such as smoking and obesity. Few studies have shown examined the correlation between chronic inflammation and anthropomorphic measurements of obesity and their influence on PCa [12].

Research in this context remains difficult due to many confounding factors such as physical activity, smoking status, obesity and the association of these with diseases such as diabetes which are all implicated in cancer and inflammation [13]. In this present study, we analyzed the interaction between NLR and PLR as widely available serum markers of systemic inflammation and csPCa while considering potential confounding factors such as obesity and smoking.

## Materials and methods

We undertook a post hoc substudy of the prospective RADICAL-PC (Role of Androgen-Deprivation Therapy In CArdiovascular Disease—A Longitudinal Prostate Cancer NCT03127631) [14] study.

### The study, endpoints and exclusion criteria

RADICAL-PC is a prospective study in 55 academic and community cancer sites in 7 countries. It includes assessment of cardiovascular risk factors in men with PCa. Recruitment started on 2015-10-21 and ended on 2023-10-18. For this substudy, we included all patients from two participating sites where complete blood count (CBC) was recorded at study enrollment. The purpose of this study was to analyze the relationship between these blood markers associated with systemic inflammation and csPCa defined as Gleason ≥ 4 + 3 at diagnosis. We decided on this definition because it represents a clear meaningful definition of clinically significant disease with a prostate cancer-specific mortality rate at 10 years of 24% [15].

This specific study was reviewed and approved by the Hamilton Integrated Research Ethics Board (CTO-0743 Ontario, Canada) before the study began. The study was conducted according to the Declaration of Helsinki. All RADICAL-PC participants provided written informed consent. This study adheres to CONSORT guidelines.

**Fig. 1:**
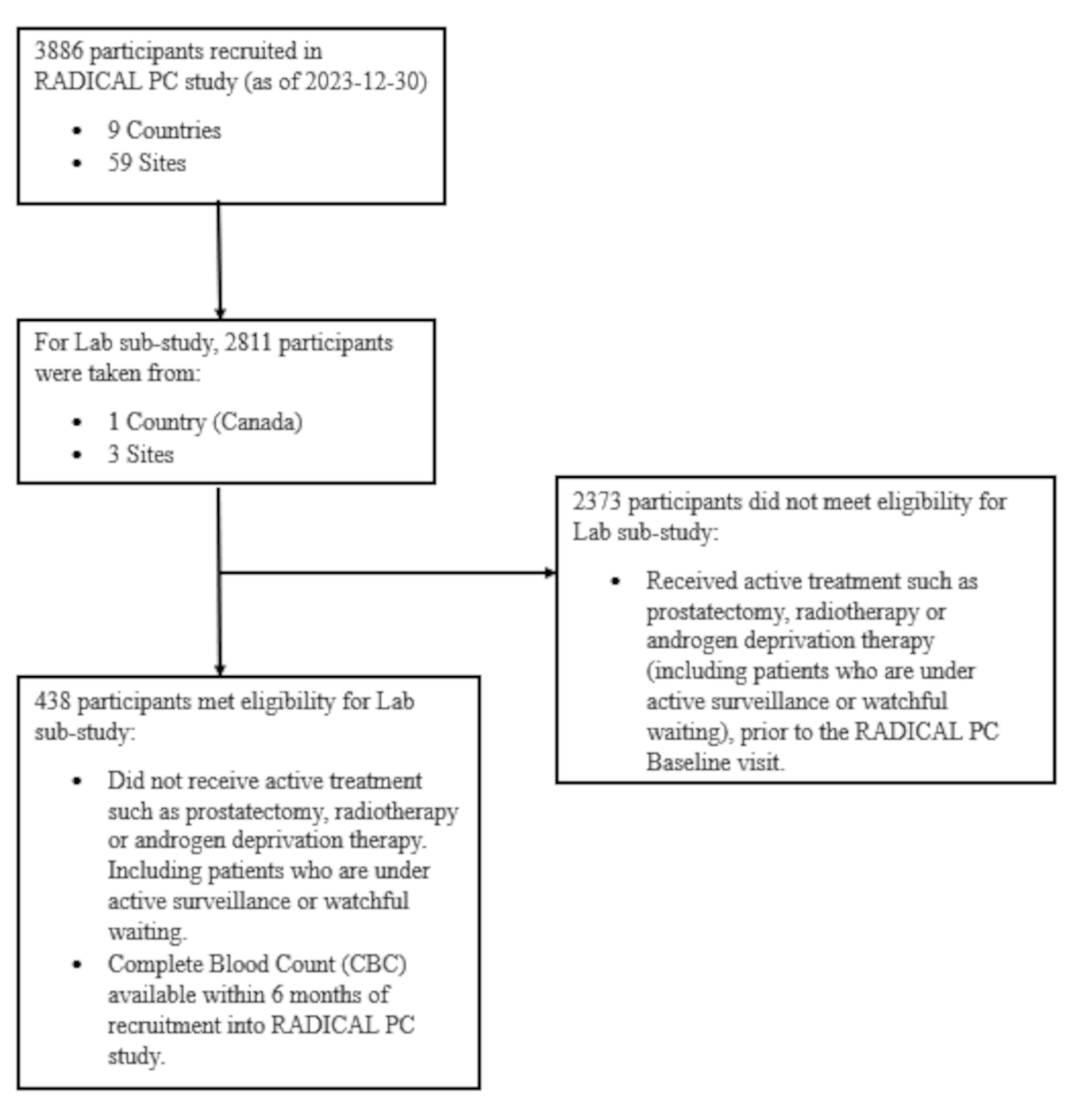
CONSORT flow diagram

In this substudy, we excluded patients who had not received active treatment, such as prostatectomy, radiotherapy, or androgen deprivation therapy, at the time of blood collection. The primary outcome was csPCa at diagnosis, defined as Gleason ≥ 4 + 3 (ISUP grade 3) disease. A CONSORT Flow Diagram is shown in Fig. 1.

### Analyzed serum markers of inflammation

We analyzed the following serum markers associated with inflammation for their influence on csPCa: neutrophil count, lymphocyte count, platelet count, hemoglobin; neutrophil-lymphocyte ratio (NLR), platelet-lymphocyte ratio (PLR), plateletcrit (PLT x MPV/ 10 000). Although there is no evidence that the platelet count or PLR are associated with elevated levels of platelet-related factors, platelets are known to release several factors mediating inflammation and promoting cancer-cell replication such as Platelet derived growth factor (PDGF), Transforming growth factor-β (TGF-β) and vascular endothelial growth factor (VEGF) [16].

### Statistical analysis

Differences between patients with csPCa and non-csPCA were evaluated using the chi-square test and analysis of variance (for normally distributed variables) or the Kruskal-Wallis test (for non-normally distributed variables). Multivariable logistic regression analysis adjusted for smoking, hypertension, diabetes, cardiovascular disease and either body-mass index (BMI) or waist-to hip ratio (WHR) was used to predict for csPCa. The threshold for statistical significance was set at 0.05. We used STATA 18, College Station, Texas, USA for statistical analysis.

## Results

Of the 443 patients enrolled at the time of data analysis. There were 51 with Gleason 6, 236 Gleason 3 + 4, 93 Gleason 4 + 3, 63 Gleason 8 or higher. Using the D’Amico risk scale, there were 27 at low risk, 250 intermediate risk and 168 high risk. A total of 156 (35%) patients had csPCa (Gleason score ≥ 4 + 3) at the time of biopsy. Patients with csPCA were significantly older than patients with a lower Gleason grade (68 ± 7 vs. 65 ± 8, *p* < 0.0001. The mean BMI didn’t differ between both groups (*p* = 0.25) and was close to the definition for obese (< 30 kg/m^2^) with 28.3 ± 3.9 kg/m^2^ and 28.8 ± 4.8 kg/m^2^, resp. (Table 1).

**Table 1:** Patient characteristics (*n* = 443).

Characteristic	Gleason < 4 + 3 (*N* = 287)	Gleason ≥ 4 + 3 (*N* = 156)	*p*-value
Age, years	65 ± 7	68 ± 7	< 0.0001
PSA at enrolment (ng/mL)	6.7 (4.5–9.9)	9.1 (5.8–14)	0.0001
Hb, g/dL	14.6 ± 1.3	14.4 ± 1.6	0.16
Neutrophil count (x10^6^/mL)	4.4 ± 1.8	4.4 ± 1.6	0.79
Lymphocyte count (x10^6^/mL)	1.8 ± 0.6	1.8 ± 0.6	0.97
Platelet count (x10^6^/mL)	224 ± 52	217 ± 48	0.13
Platelet volume (fL)	10.0 ± 1.1	10.1 ± 1.1	0.59
Plateletcrit ^1^	2224 ± 514	2165 ± 444	0.27
NLR^2^	2.66 ± 1.45	2.61 ± 1.37	0.76
PLR^3^	137 ± 54	131 ± 51	0.24
Albumin (g/L)	43.2 ± 4.3	42.6 ± 4.2	0.27
BMI^4^	28.3 ± 3.9	28.8 ± 4.8	0.25
WHR	1.00 ± 0.07	1.00 ± 0.06	0.71
Tobacco Never Former Current	134 (47) 129 (45) 23 (8)	79 (51) 63 (40) 14 (9)	0.63
Diabetes	30 (10)	30 (19)	0.010
Hypertension	120 (42)	65 (42)	0.98
Baseline CVD	45 (16)	33 (21)	0.15

CVD defined as the presence of peripheral arterial disease, coronary artery disease, cerebrovascular disease or stroke, heart failure or atrial fibrillation.

BMI = Body mass index, Hb = hemoglobin, NLR = Neutrophil-to-lymphocyte ratio, PLR = Platelet-to-lymphocyte ratio, PSA = XXX, WHR = XXX.

^1^ (PLT x MPV/ 10 000).

### Analysis of serum markers of inflammation

None of the factors such as plateletcrit, NLR, and PLR were significantly different between both groups (see Table 1).

In multivarible analysis, when adjusting for smoking status, BMI, diabetes, hypertension and history of CVD, there was no statistically significant predictive value for any of the tested variables (Table 2).

**Table 2:** Odds ratios (95% CI) for Gleason ≥ 4 + 3 adjusted for tobacco, **BMI**, diabetes, hypertension and past history of CVD

Characteristic	OR (95% CI)	*p*-value
Platelet count, per 20 × 10^9^/L	0.91 (0.81–1.02)	0.11
Plateletcrit, 100	0.96 (0.89–1.03)	0.24
NLR	0.86 (0.72–1.02)	0.079
PLR	0.995 (0.989-1.00)	0.063

Plateletcrit (PLT x MPV/ 10 000)

neutrophils to lymphocytes ratio (NLR)

platelet-lymphocyte ratio (PLR),

When adjusted for waist-to-hip ratio instead of BMI (Table 3), the results remained very similar.

**Table 3:** Odds ratios (95% CI) for Gleason ≥ 4 + 3 adjusted for tobacco, **waist-hip ratio**, diabetes, hypertension and past history of CVD

Characteristic	OR (95% CI)	*p*-value
Platelet count, per 20 × 10^9^/L	0.91 (0.81–1.02)	0.11
Plateletcrit, 100	0.96 (0.89–1.03)	0.24
NLR	0.86 (0.72–1.02)	0.079
PLR	0.995 (0.990-1.000)	0.056

Plateletcrit (PLT x MPV/ 10 000)

neutrophils to lymphocytes ratio (NLR)

platelet-lymphocyte ratio (PLR)

The serum factors of inflammation were not useful in predicting clinically significant cancers in the receiver operating characteristic curve (ROC) analysis. All factors had an AUC of ≤ 0.6.

### Alternative endpoint PSA > 10 ng/mL

In an exploratory analysis with a PSA > 10 ng/mL as the definition of csPCa instead of Gleason of ≥ 4 + 3 in univariate and multivarible logistic regression analysis, none of the factors in Tables 2 and 3 showed any significant association.

## Discussion

In this present study we could not find that csPCa defined as Gleason score ≥ 4 + 3 was associated with PLR, NLR plateletcrit or platelet count or BMI or WHR and smoking which are all associated with chronic inflammation. Results of studies investigating an association of chronic inflammation and PCa have not been consistent. Inflammation has been shown to be associated with more aggressive disease and with a higher rate of biochemical progression. Blood markers of systemic inflammation such as NLR and PLR appear to have a significant prognostic value [10]. The clinical utility and application of our findings to primary care physicians is limited. This is essentially a negative study that could not find an association between csPCa and the serum markers of inflammation. Therefore, we cannot advocate for anti-inflammatory measures such as plant-based or vegetable-forward diets (Mediterranean diet), which have been shown to have a small benefit [17].

Studies investigating an association between obesity and PCa vary considerably [18–20]. Anthropometric measurements other than BMI such as WHR have been shown to be positively linked to PCa occurrence [18]. A recent publication in Canadian men showed that smoking status modified the risk of PCa in univariate analysis in obese men (BMI > 30 kg/m^2^) who were former smokers [21].We found that both the platelet-count and plateletcrit were not associated with csPCa. Platelets can be associated with general mortality. In a general register-based cohort study of Danish adults, platelets had a U-shaped relationship with overall mortality [22]. Therefore, both low and high platelet counts were associated with an increased mortality risk. In this study an association between platelet count and cancer diagnosis was found. Platelets regulate a variety of inflammatory mechanisms and release different cytokines that have mitogenic and inflammatory properties [23]. Not only PCa, but also CVD is associated with platelet count, which depends itself on body-fat distribution [24]. Platelets can secrete inflammatory mediators such as vascular endothelial growth factor (VEGF) and platelet-derived growth factor (PDGF) that have themselves an influence on cancers [7]. In the first stages of cancerous transformation, platelet recruitment is increased to repair leaky blood vessels, causing thrombocytosis, a process that has been shown to be particularly present in PCa while anti-androgen therapy has been shown to slow down this process [25, 26].

Because of insufficient follow-up, the influence of treatment outcome of the inflammatory markers in our patients could not be investigated. The white blood count and neutrophil count have been shown to be independent prognostic factors of OS in patients treated with radiotherapy for localized PCa [27, 28]. Higher PLR-levels have been shown to be associated with worse clinical outcomes. A lower PLR was associated with better overall survival (OS) and better–metastasis-free survival [29]. In an analysis of 374 patients treated with radiotherapy, it was found that a PLR ≥ 190 was as an independent prognostic factor for worse metastasis-free survival, even when adjusted for CRP in a subgroup who had a CRP available. In a study of 290 patients treated with primary androgen deprivation therapy (ADT), it was found that the best cut-off value for PLR was 117.58. This value was a significant prognostic factor for progression-free survival, cancer-specific survival and overall survival [30]. The interaction between platelets and neutrophils is also of interest. Platelets are known to be in a hyperactive state in malignant disease, adhere to neutrophils and promote metastasis. Neutrophils can have antitumorigenic as well as pro-tumerigenic activity [31]. Others have shown that monocyte fraction and the monocyte-to-lymphocyte ratio (MLR) were significantly associated with high Gleason score prostate cancer [32].

Reducing systemic inflammation has the potential for important general health benefits in patients with PCa. This can be done for example through diet and physical activity [18, 33, 34]. Another potential options are drug-treatments to reduce inflammation such as canakinumab, an antibody targeting interleukin-1β. It has shown reduced overall cancer mortality compared to placebo [35]. IL-1 is an inflammatory cytokine which plays a key role in carcinogenesis and tumor progression. Aspirin (acetylsalicylic acid) is another example of a drug benefiting anti-inflammatory in PCa and other cancers. It inhibits platelet prostaglandin synthesis and the ADP- and collagen-induced platelet release reaction. The Physicians’ Health Study showed that current and past regular aspirin-use was associated with a lower risk of lethal PCa [36].

PCa is a heterogeneous disease. In an analysis published in 2013 [1], it was stated that increasing evidence suggest am association between obesity and an elevated incidence of aggressive PCa. It is difficult to clearly associate PCa with obesity defined as increased BMI, although several studies have shown a consistent association of aggressive PCa with obesity. An analysis of the Health Professionals Follow-up Study showed that BMI wasn’t significantly associated with the risk of lethal PCa [37] and a systematic review of obesity as a prognostic factor found that obesity was associated with increased PC-specific mortality and all-cause mortality [38]. There are studies showing that markers of abdominal obesity such as waist circumference (WC) and WHR are better markers for obesity than BMI [39]. Further elucidation of the relationships between obesity, inflammation, and its serum-markers such as platelets with cancer and other diseases such as diabetes is needed.

The strength of our study is that the participating patients were from a well-characterized population from a multicenter, prospective study. Limitations are the limited number of patients (*n* = 438) with only about 35% with csPCa. A larger sample size could have detected a smaller, statistically significant difference for a certain marker. There are many different definitions of csPCa. We predefined our endpoint prior to the analysis. We admit that using different definitions or using a combination clinical or radiological stage or percentage of positive biopsies could have changed our results. However, this is a prospective multicenter study with ongoing accrual which will increase the power of this study. This is a pragmatic trial whose explanatory results aim to test the hypothesis of an association of PCa with readily available blood markers of inflammation. Studies including Il-6 and CRP, markers that were not available for this study, would have greatly enhanced the value of our data. Our substudy includes only a small number of the patients enrolled in the RADICAL-PC study which included several thousand patients, because few had a CBC done. This could have introduced an unknown bias. Our study didn’t investigate the prognostic significance of inflammatory markers. This study was not designed to estimate prediction effects. The logistic regression model can only estimate these associations. The design of our study only allowed for the identification of associations, not predictions. Unfortunately, this was a multicenter study with a different access to MRI. We were unable to use the stage on MRI as a definition for csPCa.

In conclusion, PLR, NLR plateletcrit and platelet count were not associated with Gleason score ≥ 4 + 3 disease, nor were factors associated with chronic inflammation, such as obesity and smoking. More sophisticated markers of inflammation are needed to further investigate the interaction between inflammation and prostate cancer.

## Electronic supplementary material

Below is the link to the electronic supplementary material.

Supplementary Material 1: CONSORT-2010-Checklist.

## Data Availability

The data will be made available on request for the sole purpose of replicating the study’s findings and that requests must be sent to the study Steering Committee, whose role includes data access external to the study, care of the study PI’s, Darryl Leong or Jehonathan Pinthus. Specifically, we will require a proposal to describe the rationale for the request and the statistical methods that are proposed. The proposal must be submitted to both the study Publications and Steering Committees for approval. These committees will ensure that the proposal does not conflict with ongoing analyses and the quality of the proposal. Once approved, the requesting party will be required to enter a contractual agreement with the study sponsor, which will include the terms and conditions of data release as well as the costs incurred in the proposal review and data curation. These costs will be invoiced to the requesting party. Please contact steve.agapay@phri.ca for this matter.

## Abbreviations

(PCa): Prostate cancer
(NLR): Neutrophil indexed to lymphocyte count
(PLR): Platelet counts indexed to lymphocyte count
